# Supplementary material for: From sequence to enzyme mechanism using multi-label machine learning
Source: BMC Bioinformatics. 2014 May 19;15:150. doi: 10.1186/1471-2105-15-150 (PMC4229970; doi:10.1186/1471-2105-15-150)
Supplement: Additional file 2 — Java code of ml2db. Additional file ml2db_code.tar.gz contains the Java source code to run the multi-label machine learning experiments and save the results to database. The code’s Javadoc is included. [file 1471-2105-15-150-S2.zip › additional file 2/ml2db/ml2db/doc/main/ml2db/mulan/learn/class-use/MulanLearner.html]

Uses of Class main.ml2db.mulan.learn.MulanLearner


JavaScript is disabled on your browser.


- Overview
- Package
- Class
- Use
- Tree
- Deprecated
- Index
- Help

- Prev
- Next

- Frames
- No Frames

- All Classes

## Uses of Class main.ml2db.mulan.learn.MulanLearner

- Packages that use MulanLearner

  | Package | Description |
  | --- | --- |
  |  |  |
  | --- | --- |
  | main.ml2db.al |  |
  | main.ml2db.al.database |  |
  | main.ml2db.al.learning |  |
  | main.ml2db.mulan.learn |  |
  | main.ml2db.mulan.learn.crosseval |  |
  | main.ml2db.mulan.learn.traintest |  |
  | main.ml2db.mulan.predict |  |
  | test.ml2db.mulan.learn |  |
  | test.ml2db.mulan.learn.traintest |  |
  | test.ml2db.mulan.predict |  |
- - ### Uses of MulanLearner in main.ml2db.al

    Methods in main.ml2db.al that return MulanLearner

    | Modifier and Type | Method and Description |
    | --- | --- |
    |  |  |
    | --- | --- |
    | `MulanLearner` | ActiveLearning.`getTrainedLearner()` |

    Methods in main.ml2db.al with parameters of type MulanLearner

    | Modifier and Type | Method and Description |
    | --- | --- |
    |  |  |
    | --- | --- |
    | `void` | ActiveLearning.`setTrainedLearner(MulanLearner mulanLearner)` |
  - ### Uses of MulanLearner in main.ml2db.al.database

    Methods in main.ml2db.al.database with parameters of type MulanLearner

    | Modifier and Type | Method and Description |
    | --- | --- |
    |  |  |
    | --- | --- |
    | `uk.ac.standrews.utils.main.database.TableRow` | AlExperimentsTable.`createRow(int labelledArffId, int unlabelledArffId, java.lang.String startTime, MulanLearner learner, java.lang.String distanceFunction, java.lang.String utilityFunction, uk.ac.standrews.utils.main.database.TableManager tableManager)` Create a row with the experiment details |
  - ### Uses of MulanLearner in main.ml2db.al.learning

    Methods in main.ml2db.al.learning that return MulanLearner

    | Modifier and Type | Method and Description |
    | --- | --- |
    |  |  |
    | --- | --- |
    | `MulanLearner` | ExperimentDetails.`getAlgorithm()` |

    Constructors in main.ml2db.al.learning with parameters of type MulanLearner

    Constructor and Description

    `AlExperimenter(mulan.data.MultiLabelInstances trainSet,
    mulan.data.MultiLabelInstances testSet,
    DbManagers dbMan,
    MulanLearner learner,
    java.lang.String resultsDirectory,
    ActiveLearning experiment)`

    `ExperimentDetails(IdMultiLabelInstances unlabelled,
    MulanLearner algorithm,
    java.lang.String distanceFunction,
    DbManagers dbManagers,
    UtilityMetric utilityMetric,
    java.lang.String imagesPath)`

    With set of unlabelled instances (no arff record identifier)

    `ExperimentDetails(int labelledArffId,
    int unlabelledArffId,
    MulanLearner algorithm,
    java.lang.String distanceFunction,
    DbManagers dbManagers,
    UtilityMetric utilityMetric,
    java.lang.String imagesPath)`

    Both labelled and unlabelled arff record identifier.

    `ExperimentDetails(int unlabelledArffId,
    MulanLearner algorithm,
    java.lang.String distanceFunction,
    DbManagers dbManagers,
    UtilityMetric metric,
    java.lang.String imagesPath)`

    Only Unlabelled arff record identifier.
  - ### Uses of MulanLearner in main.ml2db.mulan.learn

    Methods in main.ml2db.mulan.learn that return MulanLearner

    | Modifier and Type | Method and Description |
    | --- | --- |
    |  |  |
    | --- | --- |
    | `static MulanLearner` | MulanLearners.`getBinaryRelevanceWrappedClassifier(weka.classifiers.Classifier classifier, java.lang.String classifierName)` |
    | `static MulanLearner` | MulanLearners.`getBPMLLBackPropagation()` |
    | `static MulanLearner` | MulanLearners.`getBRk1NN()` (Binary Relevance Nearest Neighbours) |
    | `static MulanLearner` | MulanLearners.`getBRk1NNAL()` (Binary Relevance Nearest Neighbours) |
    | `static MulanLearner` | MulanLearners.`getBRk1NNALJaccard()` (Binary Relevance Nearest Neighbours) |
    | `static MulanLearner` | MulanLearners.`getBRk1NNALJaccardEuclidean()` Binary Relevance Nearest Neighbours with jaccard+euclidean distance |
    | `static MulanLearner` | MulanLearners.`getBRk1NNManhattan()` Binary Relevance Nearest Neighbours learner (Active Learning version) with Manhattan distance instead of default Euclidean distance. |
    | `static MulanLearner` | MulanLearners.`getBRk2NNManhattan()` |
    | `static MulanLearner` | MulanLearners.`getBRk5NNAL()` (Binary Relevance Nearest Neighbours) |
    | `static MulanLearner` | MulanLearners.`getBRkNN(int neighbours)` public static MulanLearner getBRk1NNAL() { return MulanLearner.getBRkNNAL(MulanLearners.K1); } |
    | `static MulanLearner` | MulanLearners.`getBRkNNAL(int neighbours)` |
    | `static MulanLearner` | MulanLearners.`getEnsembleOfClassifierChains(int numberOfModels)` The Classifier Chain model ( CC ) involves \| L \| binary classifiers as in BM . |
    | `static MulanLearner` | MulanLearners.`getHierarchicalMultilabel(mulan.classifier.MultiLabelLearnerBase multiLabelClassifier, java.lang.String classifierName)` |
    | `static MulanLearner` | MulanLearners.`getHomer()` |
    | `static MulanLearner` | MulanLearners.`getLabelPowersetJ48C45()` From Wikipedia: C4.5 is an algorithm used to generate a decision tree developed by Ross Quinlan. |
    | `static MulanLearner` | MulanLearners.`getLabelPowersetSMO()` |
    | `static MulanLearner` | MulanLearners.`getLabelPowersetSMOReg()` |
    | `static MulanLearner` | MulanLearners.`getLabelPowersetWrappedClassifier(weka.classifiers.Classifier classifier, java.lang.String classifierName)` |
    | `MulanLearner` | MulanSerializer.`getLearner()` |
    | `static MulanLearner` | MulanLearners.`getNaiveBayesBinaryRelevance()` |
    | `static MulanLearner` | MulanLearners.`getNaiveBayesLabelPowerset()` |
    | `static MulanLearner` | MulanLearners.`getOneRule()` |
    | `static MulanLearner` | MulanLearners.`getPerceptron()` |
    | `static MulanLearner` | MulanLearners.`getRakel_J48_C45()` From Wikipedia: C4.5 is an algorithm used to generate a decision tree developed by Ross Quinlan. |
    | `static MulanLearner` | MulanLearners.`getRakelWrappedClassifier(mulan.classifier.MultiLabelLearner multiLabelClassifier, java.lang.String classifierName)` RAKEL-MLKNN (RAndom k-labELsets) // failed not enough labels in metadata |
    | `static MulanLearner` | MulanLearners.`getZeroRule()` public static MulanLearner getWrappedClassifier(TransformationBasedMultiLabelLearner wrapper, String wrapperName, Classifier classifier, String classifierName){ TransformationBasedMultiLabelLearner wrapped = new TransformationBasedMultiLabelLearner(classifier); wrapper.setDebug(true); return new MulanLearner(MulanLearner.getName(wrapper), wrapperName + "\_" + classifierName, wrapped); } |

    Methods in main.ml2db.mulan.learn that return types with arguments of type MulanLearner

    | Modifier and Type | Method and Description |
    | --- | --- |
    |  |  |
    | --- | --- |
    | `static java.util.Vector<MulanLearner>` | MulanLearners.`getLearners()` |

    Methods in main.ml2db.mulan.learn with parameters of type MulanLearner

    | Modifier and Type | Method and Description |
    | --- | --- |
    |  |  |
    | --- | --- |
    | `static java.lang.String` | MulanSerializer.`run(int trainArffId, MulanLearner learner, DbManagers dbMan, java.lang.String folderForModel)` |

    Constructors in main.ml2db.mulan.learn with parameters of type MulanLearner

    Constructor and Description

    `MulanSerializer(MulanArffRecord arffRecord,
    MulanLearner learner,
    java.lang.String directoryForSerializedModel)`
  - ### Uses of MulanLearner in main.ml2db.mulan.learn.crosseval

    Methods in main.ml2db.mulan.learn.crosseval that return MulanLearner

    | Modifier and Type | Method and Description |
    | --- | --- |
    |  |  |
    | --- | --- |
    | `MulanLearner` | CrossExperimenter.`getLearner()` |

    Method parameters in main.ml2db.mulan.learn.crosseval with type arguments of type MulanLearner

    | Modifier and Type | Method and Description |
    | --- | --- |
    |  |  |
    | --- | --- |
    | `static void` | CrossExperimenter.`run(int[] arffFilesIds, java.lang.String resultsDbPropsPath, int folds, java.util.Vector<MulanLearner> learners, boolean savePredictions, boolean saveTrueNegatives, boolean stratify, int numberOfCrossEval)` Run a cross-evaluation of several ARFF files (using one or several algorithms, as needed) |

    Constructors in main.ml2db.mulan.learn.crosseval with parameters of type MulanLearner

    Constructor and Description

    `CrossExperimenter(DbManagers dbMan,
    int trainArffRecordId,
    MulanLearner learner,
    java.lang.String resultsFolder)`

    `CrossExperimenter(IdMultiLabelInstances trainSet,
    MulanLearner learner,
    DbManagers dbMan,
    java.lang.String resultsFolder)`
  - ### Uses of MulanLearner in main.ml2db.mulan.learn.traintest

    Methods in main.ml2db.mulan.learn.traintest with parameters of type MulanLearner

    | Modifier and Type | Method and Description |
    | --- | --- |
    |  |  |
    | --- | --- |
    | `static void` | TrainTestFullRun.`fullRun(DbManagers dbs, boolean filtered, boolean serialise, boolean predict, boolean saveTrueNegatives, int train, int test, MulanLearner learner)` Executes: 1. |
    | `static void` | TrainTestExperimenterSerialized.`runWithSerializedTrainedModel(DbManagers arffStoreAndResultsDbManagers, int trainArffRecordId, int testArffRecordId, MulanLearner learner, java.lang.String trainedModelFile, java.lang.String resultsFilesDirectory, boolean predict, boolean saveTrueNegatives)` |
    | `static java.lang.String` | TrainTestExperimenterSerialized.`trainAndSerialize(int trainArffRecordId, MulanLearner learner, DbManagers dbManagers, java.lang.String directoryForSerializedModel)` |

    Constructors in main.ml2db.mulan.learn.traintest with parameters of type MulanLearner

    Constructor and Description

    `TrainTestExperimenter(DbManagers dbMan,
    int trainArffRecordId,
    int testArffRecordId,
    MulanLearner learner,
    java.lang.String resultsDirectory)`

    `TrainTestExperimenter(IdMultiLabelInstances trainSet,
    IdMultiLabelInstances testSet,
    DbManagers dbManagers,
    MulanLearner learner,
    java.lang.String resultsDirectory)`

    `TrainTestExperimenterSerialized(DbManagers dbMan,
    int trainArffRecordId,
    int testArffRecordId,
    MulanLearner learner,
    java.lang.String trainedModelFile,
    java.lang.String resultsDirectory)`
  - ### Uses of MulanLearner in main.ml2db.mulan.predict

    Constructors in main.ml2db.mulan.predict with parameters of type MulanLearner

    Constructor and Description

    `PredictionsSaver(uk.ac.standrews.utils.main.database.DbManager resultsDbManager,
    int trainArffId,
    int testArffId,
    IdMultiLabelInstances fullTrainSet,
    IdMultiLabelInstances fullTestSet,
    mulan.data.MultiLabelInstances trainSet,
    mulan.data.MultiLabelInstances testSet,
    MulanLearner baseLearner,
    mulan.classifier.MultiLabelLearner trainedLearner,
    boolean saveTrueNegatives,
    int fold)`
  - ### Uses of MulanLearner in test.ml2db.mulan.learn

    Methods in test.ml2db.mulan.learn that return MulanLearner

    | Modifier and Type | Method and Description |
    | --- | --- |
    |  |  |
    | --- | --- |
    | `static MulanLearner` | MulanLearnerTest.`getMulanLearnerOneR()` |
    | `static MulanLearner` | MulanLearnerTest.`getMulanLearnerZeroR()` |
  - ### Uses of MulanLearner in test.ml2db.mulan.learn.traintest

    Methods in test.ml2db.mulan.learn.traintest with parameters of type MulanLearner

    | Modifier and Type | Method and Description |
    | --- | --- |
    |  |  |
    | --- | --- |
    | `static TrainTestExperimenter` | TrainTestExperimenterTest.`getMulanTrainTestExperById(int trainId, int testId, MulanLearner learner)` |
  - ### Uses of MulanLearner in test.ml2db.mulan.predict

    Methods in test.ml2db.mulan.predict with parameters of type MulanLearner

    | Modifier and Type | Method and Description |
    | --- | --- |
    |  |  |
    | --- | --- |
    | `static PredictionsSaver` | PredictionsSaverTest.`getSaver(java.lang.String trainDatasetName, java.lang.String trainProps, java.lang.String testDatasetName, java.lang.String testProps, MulanLearner learner)` |

- Overview
- Package
- Class
- Use
- Tree
- Deprecated
- Index
- Help

- Prev
- Next

- Frames
- No Frames

- All Classes
